# Supplementary material for: Identification of photoperiod-induced specific miRNAs in the adrenal glands of Sunite sheep (Ovis aries)
Source: Front Vet Sci. 2022 Jul 22;9:888207. doi: 10.3389/fvets.2022.888207 (PMC9354845; doi:10.3389/fvets.2022.888207)
Supplement: Supplementary file 1 [file Data_Sheet_1.ZIP › Supplementary file/miRDeep2 file.zip!/miRDeep2 file/Know_Structure/SP42A2/oar-mir-99a.pdf]

miRBase precursor : oar-mir-99a
Total read count : 898313
oar-miR-99a read count : 897598
remaining reads : 715

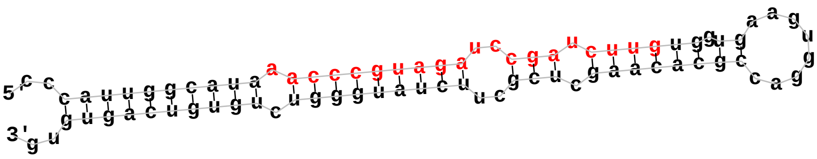

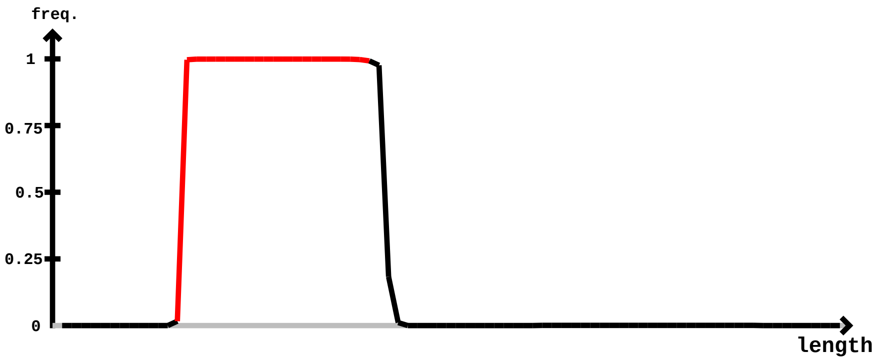

oar-miR-99a

5'- cccauuggcauaaacccguagauccgaucuuguggugaaguggaccgcacaagcucgcuucuaugggucugugucagugug -3'	exp		
..((((((((((.(((((((((..(((.((((((.((........)))))))).)))..))))))))).)))))))))))..	reads	mm	sample
..........uaaacccguagauccgaucu...	1	0	seq
..........uaaacccguagauccgaucuugu..	4	0	seq
...........aaacccguagauccgauc..	4	0	seq
...........aaacccguagauccgaucu...	22	0	seq
...........aaacccguagauccgaucuu..	70	0	seq
...........aaacccguagauccgaucuug...	223	0	seq
...........aaacccguagauccgaucuugu..	14075	0	seq
...........aaacccguagauccgaAcuugug...	1	1	seq
...........aaacccguagauccgaucuugug...	315	0	seq
...........aaacccguagauccgaucuugugg..	4	0	seq
............aacccguagauccgauc..	211	0	seq
............aacccguagauccgaAc..	7	1	seq
............aacccguagauccgaAcu...	119	1	seq
............aacccguagauccgaucu...	1288	0	seq
............aacccguagauccgaAcuu..	234	1	seq
............aacccguagauccgaucuu..	4152	0	seq
............aacccguagauccgaAcuug...	1830	1	seq
............aacccguagauccgaucuug...	12618	0	seq
............aacccguagauccgaAcuugu..	27804	1	seq
............aacccguagauccgaucuugu..	669385	0	seq
............aacccguagauccgaAcuugug...	37340	1	seq
............aacccguagauccgaucuugug...	115684	0	seq
............aacccguagauccgaucuugugg..	9553	0	seq
............aacccguagauccgaAcuugugg..	363	1	seq
............aacccguagauccgaucuuguggu...	23	0	seq
............aacccguagauccgaAcuuguggu...	2	1	seq
.............acccguagauccgaucu...	2	0	seq
.............acccguagauccgaucuu..	6	0	seq
.............acccguagauccgaAcuu..	3	1	seq
.............acccguagauccgaAcuug...	27	1	seq
.............acccguagauccgaucuug...	4	0	seq
.............acccguagauccgaucuugu..	808	0	seq
.............acccguagauccgaAcuugu..	336	1	seq
.............acccguagauccgaucuugug...	142	0	seq
.............acccguagauccgaAcuugug...	788	1	seq

oar-miR-99a

```
cccauuggcauaaacccguagauccgaucuuguggugaaguggaccgcacaagcucgcuucuaugggucugugucagugug
.............acccguagauccgaAcuugugg..............................................	18	1	seq
.............acccguagauccgaucuugugg..............................................	16	0	seq
.............acccguagauccgaucuuguggu.............................................	1	0	seq
.............acccguagauccgaAcuuguggu.............................................	1	1	seq
..............cccguagauccgaucuugu................................................	21	0	seq
..............cccguagauccgaucuugugg..............................................	1	0	seq
...............ccguagauccgaucuug.................................................	1	0	seq
...............ccguagauccgaucuugu................................................	42	0	seq
...............ccguagauccgaAcuugu................................................	1	1	seq
...............ccguagauccgaAcuugug...............................................	1	1	seq
...............ccguagauccgaucuugug...............................................	9	0	seq
................cguagauccgaucuugu................................................	23	0	seq
................cguagauccgaucuugug...............................................	13	0	seq
................cguagauccgaAcuugug...............................................	2	1	seq
................................................acaagcucgcuucuauggguc............	1	0	seq
.................................................caagcucgcuucuauggguc............	3	0	seq
.................................................caagcucgcuucuaugggucu...........	7	0	seq
.................................................caagcucgcuucuaugggucug..........	16	0	seq
.................................................caagcucgcuucuaugggucugu.........	658	0	seq
..................................................aagcucgcuucuauggguc............	1	0	seq
..................................................aagcucgcuucuaugggucugu.........	29	0	seq
```
